# [Un]met Need and [Un]wanted Family Planning: A Cross‐Sectional Study Among Women in Argentina, Ghana, and India Examining Characteristics, Reasons, and Alignment With Fertility Desires

**DOI:** 10.1111/sifp.70035

**Published:** 2025-09-16

**Authors:** Jewel Gausman, Niranjan Saggurti, Richard Adanu, Delia A. B. Bandoh, Mabel Berrueta, Suchandrima Chakraborty, Ernest Kenu, Nizamuddin Khan, Ana Langer, Nigri Carolina, Magdalene A. Odikro, Veronica Pingray, Sowmya Ramesh, Paula Vázquez, Caitlin R. Williams, R. Rima Jolivet

## Abstract

Unwanted family planning often refers to fertility desires as a proxy for contraceptive desire and lacks alignment with the tenets of person‐centered care. We construct a person‐centered measure of unwanted family planning by asking women whether they wanted to use a method, examine its alignment with the fertility‐derived measure, and describe the characteristics of women with unwanted family planning and reasons women state for not wanting to use a method. We conducted a cross‐sectional study of women aged 15–49 in Argentina, Ghana, and India. Data were collected on stated desire to use contraception and basic sociodemographic characteristics. Fertility desire was collected using the standard Demographic and Health Survey questionnaire. In total, 4794 women were included in our study. Among women using a method, 2.5 percent (*n* = 53) of women had unwanted family planning, with 4.2 percent in Ghana, 2.2 percent in Argentina, and 2.0 percent in India. Most unwanted family planning (85.2 percent, *n* = 23) occurred among women who did not want a child within the next nine months. Sexual infrequency was the most common reason behind a lack of desire to use a method. Our results highlight the substantial differences found between classifying women's contraceptive needs from a person‐centered versus a fertility‐derived approach.

## INTRODUCTION

The concept of unwanted family planning is rooted within a larger framework of reproductive coercion, which is defined as intentional interference with another's reproductive autonomy or pregnancy outcomes. Most research on reproductive coercion focuses on actions, primarily within the context of an intimate partnership, that are taken to undermine one's use of contraception, apply pressure to become pregnant, or exert control over the termination or continuation of a pregnancy (Miller, Decker, et al. 2010; Miller et al. 2007; Miller, Jordan, et al. 2010). Some studies have defined it as a form of intimate partner violence, while others have found a strong association between it and other forms of abuse (Wood et al. 2023). In the 2018 roll‐out of the Phase 8 Model Women's Questionnaire, the Demographic and Health Survey (DHS) included a single item on reproductive coercion: “Has your (husband/partner) or any other family member ever tried to force or pressure you to become pregnant when you did not want to become pregnant?” (DHS 2016)

Most research to date on reproductive coercion offers limited insight into situations where pressure is put on individuals to use contraception (Grace and Anderson 2018; Wood et al. 2023) either by the health service delivery system or in interpersonal relationships. In response to this gap, Canning and Karra have explored the concept of unwanted family planning, defined as contraceptive use among women who desire to have a child in the next nine months (Canning and Karra 2023). Their study, including data from 56 low‐ and middle‐income countries, estimates the prevalence of unwanted family planning to be approximately 2.1 percent globally.

The definition of unwanted family planning proposed by Canning and Karra focuses on the discordance between a person's contraceptive behavior and their fertility desires. It is the inverse of the concept of unmet need, which refers to the absence of contraceptive use among women who wish to avoid pregnancy. Both measures assume that one's fertility desires are a proxy for their desired contraceptive use (aka “need”). Since the 1994 Cairo Convention, advocates for a rights‐based and feminist approach to sexual and reproductive health have urged the global health community to separate itself from its history of programs focused on population control (Senderowicz and Valley 2023). Yet, by continuing to equate one's future fertility desire with contraceptive need in defining these indicators, we argue that the current definition of unmet need and unwanted family planning fails to move beyond this paradigm to decouple the broader agenda of sexual and reproductive health and rights that originated in the Cairo Convention from the field's neo‐Malthusian origins.

Recent literature has called for a renewed commitment to grounding global family planning programs in human rights principles that emphasize respect for individual autonomy, values, and preferences (Hardee et al. 2014; Holt, Dehlendorf, and Langer 2017; Organization 2016; World Health Organization 2017; UNFPA 2020). Respectful, person‐centered care is responsive to individual needs, preferences, and values; externally imposing assumptions about a woman's desire for contraception runs counter to these core principles (Dang 2018; Institute of Medicine Committee on Quality of Health Care in 2001). Neither the standard definition of unmet need nor its inverse, unwanted family planning, considers a woman's direct expression of her desire, or lack thereof, for contraception. Asking women directly about their contraceptive desires would better align the indicators within a framework of person‐centered care (Gausman et al. 2025). Furthermore, it would help move the field of sexual and reproductive health beyond a focus on fertility toward a measurement framework more supportive of a holistic view of women's sexual and reproductive health.

Recognizing that the diverse, noncontraceptive‐related motivations to use, or not to use, methods of family planning are as equally valid as those related to fertility control represents an important opportunity for the field to embrace a more comprehensive, rights‐based approach to define contraceptive need. Furthermore, it presents an opportunity to the global health community to better serve populations that have been historically marginalized from family planning programs, including unmarried women, nonsexually active women, sexual and gender‐minorities, and women at the beginning or end of their reproductive years that may still have important reasons for desiring to use a contraceptive method to improve their lives beyond their fertility‐related benefits (Gausman et al. 2025; Carroll 1999). Specifically, some hormonal methods enable some women to have more predictable and less obtrusive menstruation, improve acne and other skin conditions, and address a wide range of medical conditions, including dysmenorrhea, ovarian cysts, and provide some protection against certain types of cancers (Williams et al. 2021; Schindler 2012).

Beyond their medical benefits, the sexual health aspects of contraception for women and their intimate partners are also missing from the fertility‐derived definition of contraceptive need.

Many barrier methods prevent transmission of HIV and STIs, and some contraceptive technologies under development, such as the vaginal ring, may in the future offer a delivery mechanism for microbicides (Baeten et al. 2016). Furthermore, the reliance on fertility as a proxy for contraceptive need also discounts women's sexual agency by representing outdated social norms (Gausman et al. 2025). Globally, many married and unmarried women and men have concurrent sexual partnerships, which bring with them different contexts, risks, and goals. A woman with multiple partners may want to use on‐demand contraception with one partner while attempting a pregnancy with another partner. Conversely, a woman wanting to avoid a pregnancy in an intimate relationship may have no need for contraception due to a lack of sexual exposure that has the potential to result in a pregnancy.

In our study, we move beyond a fertility‐derived definition of unwanted family planning. Instead, we redefine the concept of unwanted family planning as a woman's use of a contraceptive method despite a directly stated lack of desire to do so. By centering the woman's voice, this shift in definition acknowledges the importance of a woman's autonomy and her explicit preferences regarding contraception, thus moving toward a more person‐centered view of sexual and reproductive health and rights. Using the revised definition, we first describe the prevalence of unwanted family planning in the study sample across three diverse low‐ and middle‐income countries. Second, we compare the revised indicator with the previously proposed version by comparing the proportion of women who fall into different categories of contraceptive need. Third, we compare the sociodemographic and contraceptive use profiles between women with unwanted versus wanted family planning using the revised definition, and we describe the reasons that women with unwanted family planning state when asked why they do not want to use a method.

## METHODS

### Study Description

This is a secondary analysis of a cross‐sectional study originally designed to validate the indicator “demand satisfied for family planning” among women aged 15–49 years in four subnational areas in three countries: Argentina, Ghana, and India, by comparing multiple approaches to its calculation. Demand satisfied refers to Sustainable Development Goal Indicator 3.7.1, which tracks the “proportion of women of reproductive age (aged 15–49 years) who have their need for family planning satisfied with modern methods.” Countries were selected through a competitive review process whereby interested research partners each from Africa, Asia, and Latin America/Caribbean submitted proposals that prioritized global geographic diversity.

### Study Design

Primary data collection was conducted in four districts/provinces per country. Districts were selected using a composite maternal health performance index to ensure diversity in sites. First, one highest and one lowest performing state/region was selected; second, one highest and one lowest performing district/province was selected within each state/region. Country‐specific adaptations were made in Argentina, including the use of terciles due to low population density and the substitution of alternative indicators due to limited geographic variation in standard maternal health metrics. More details on the selection of the geographic areas can be found in the published study protocol (Jolivet et al. 2022).

The study was designed to be representative at the subnational geographic level (district or province) in each country. Sample size calculations used the standard formula $n=\left(Z^{2}*pq\right)/d^{2},$where *Z* is the standard normal deviation, *p* is the proportion of the population using family planning, *q* is the proportion of the population not using family planning, and *d* is the degree of accuracy. The sample was further adjusted to reflect an estimated 10 percent nonresponse rate, a design effect of 2 to account for clustering, and a multiplier of 1.68 to account for the low prevalence of modern contraception in each country, yielding a final sample size of 355 women per district/province.

The study employed a random two‐stage sampling approach. For the first stage, 20 primary sampling units were randomly selected in each district/province. The sampling frame was adapted from the DHSs in Ghana and India and the Multiple Indicator Cluster Survey in Argentina. In Ghana and India, community mapping was used to identify households with women of reproductive age (15–49 years) within each primary sampling unit. From this list, at least 18 women from different households were randomly selected in each primary sampling unit, resulting in a minimum of 360 women per district and a total of 1440 women per country. In Argentina, due to low population density making household surveys unfeasible, interviews were conducted with a random sample of 360 women per primary sampling unit upon their exit from facilities providing reproductive and maternal health services. The interviews took place between October 2020 and December 2020 in Ghana, from October 2020 to March 2021 in India, and from March 2021 to June 2021 in Argentina.

### Participants

Women were included in the study if they agreed to participate and if they were aged 15–49 years at the time of the study. Written informed consent was obtained from participants ≥18 years old in Ghana and India and ≥16 years old in Argentina; parental permission and written informed assent were obtained for minors <18 years old in Ghana and India and <16 years old in Argentina.

### Data Collection

We collected basic demographic data from all participants, including wealth quintile, education, literacy, age, marital status, and place of residence. Wealth quintile was calculated by administering the country‐specific EquityTool module (EquityTool 2021), which calculates one's wealth quintile in terms of comparison to the national average.

To collect data on fertility preferences, women were asked a series of questions designed to be identical to the DHS Women's Module (The DHS Program 2015). In addition, women were also asked directly if they wanted to use a method of family planning right now. Women who did not want to use a method of family planning were asked the reason for their lack of desire to use a method from a wide range of prepopulated responses that generally pertained to lack of sexual exposure that can result in a pregnancy, desiring a pregnancy, opposition to using family planning, lack of knowledge of methods or where to access methods, and method‐related reasons or concerns.

### Analysis

Our outcome of interest was unwanted family planning. In contrast to the previously proposed definition by Canning and Karra, which is based on unmet need, we defined unwanted family planning as the use of a method of contraception among nonpregnant women who responded “no” to the question “Do you want/desire to be using a method of family planning right now?” We calculated the overall proportion of women with unwanted family planning by country.

To assess alignment between fertility preferences and demand for family planning, we compare the proportion of women who state directly wanting or not wanting to use family planning according to their fertility desires, categorized by whether they wanted to have a child within the next nine months (or now/soon), whether they wanted a child in the next two years (but more than nine months from the time of the survey), whether they were undecided on the timing of a next child or whether to have a next child, or whether they wanted no more children. We then compare the proportion of women who fall into different categories of need for family planning based on either fertility as a proxy for need or a woman's directly stated demand for contraception. For the sake of simplicity, from this point forward, we refer to unwanted family planning (indirect) as that derived from a woman's desire to have a child within in the next nine months or soon/now, and refer to unwanted family planning (direct) as that obtained from a woman's direct response indicating her desire to use or not to use contraception now.

**FIGURE 1:**
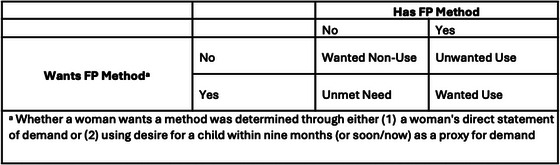
Concordance‐based approach to determining whether family planning behavior aligns with demand (Senderowicz 2020; Canning and Karra 2023)

We follow a concordance‐based approach to determine whether a woman's contraceptive behavior and preferences are aligned (Senderowicz 2020), though we vary how we establish a woman's preference. Using both the indirect and direct approach, we calculate the percentage of women with wanted family planning (current family planning users that directly state wanting to use a method), the percentage of women with unmet need for family planning (women who are not using a family planning method but directly state wanting to use a method), unmet need, unwanted use (women who are using family planning but directly state not wanting to use a method), and women with desired nonuse (women who are not using a method and directly state not wanting to use a method) (Canning and Karra 2023). In both cases, the ideal outcome is alignment between one's desire to use a method and their behavior. These definitions are illustrated in Figure 1.

Finally, we examine differences in sociodemographic characteristics and contraceptive method use between women with wanted use versus unwanted family planning (direct), and we further describe the reasons why women with unwanted family planning (direct) do not want a method. We determined the statistical significance of differences observed using chi‐squared for categorical variables and *t*‐tests for continuous variables. We report *p*‐values of less than 0.05 to determine statistical significance.

## RESULTS

In total, 4794 women were included in our study (Table 1) from Argentina (*n* = 1492), Ghana (1600), and India (*n* = 1702). The average age of participants was 29.1, with little variation across countries. The vast majority of participants were married (*n* = 3716). Ghana had the lowest proportion of women who were married (65.1 percent), and India had the highest (89 percent). In Ghana, the sample on average tended to be relatively poor, with 29.6 percent (*n* = 473) of women being in the poorest wealth quintile. Conversely, in India, the sample tended to be wealthier than the general population of the country, with 76.5 percent (*n* = 1303) of women being in the richest two wealth quintiles. Most women in Argentina (64.6 percent, *n* = 435) and India (64.5 percent, *n* = 418) stated not wanting another child. In Ghana, about half of women (*n* = 364) reported wanting a child within two years, compared to 31.9 percent (*n* = 277) in India and 15.2 percent (*n* = 98) in Argentina.

**TABLE 1:** Description of the sample in Argentina, Ghana, and India

	Argentina	Ghana	India	Total
All women (*N*)	1492	1600	1702	4794
Age, mean (SD)	28.8 (7.4)	28.3 (7.8)	30.9 (8.0)	29.1 (7.9)
Marital status, *n* (%)				
Currently married or in union	1145 (76.7)	1042 (65.1)	1529 (89.8)	3716 (77.5)
Never married	30 (2.0)	484 (30.3)	128 (7.5)	642 (13.4)
Widowed/divorced/separated	215 (21.1)	73 (4.6)	45 (2.6)	433 (9.0)
Missing	2 (0.1)	1 (0.1)	0 (0.0)	3 (0.1)
Living apart from spouse, *n* (%)				
Yes	154 (10.3)	155 (9.7)	114 (6.7)	423 (8.9)
No	987 (6.2)	881 (55.1)	1412 (83.0)	3280 (68.4)
Not currently married/in union	347 (23.3)	558 (34.9)	173 (.2)	1078 (22.5)
Missing	4 (0.3)	6 (0.4)	3 (0.2)	13 (0.3)
Highest educational attainment, *n* (%)				
None	9 (0.6)	604 (37.8)	138 (8.1)	751 (15.7)
Primary	357 (23.9)	511 (31.9)	520 (30.6)	1388 (29.0)
Secondary or higher	1119 (75.0)	473 (29.6)	1037 (60.9)	2629 (54.8)
Missing	7 (0.47)	12 (0.75)	7 (0.41)	26 (0.5)
Wealth quintile, *n* (%)				
Poorest	108 (7.24)	473 (29.6)	17 (1.0)	598 (12.47)
Poorer	308 (20.6)	349 (21.8)	86 (5.1)	743 (15.5)
Middle	442 (29.6)	310 (19.4)	293 (17.2)	1045 (21.8)
Rich	352 (23.6)	202 (12.6)	630 (37.0)	1184 (24.7)
Richest	97 (6.5)	255 (15.9)	673 (39.5)	1025 (21.4)
Missing	185 (12.4)	11 (0.7)	3 (0.2)	199 (4.2)
Currently pregnant, *n* (%)				
Yes/Don't know	368 (24.7)	362 (22.6)	237 (13.9)	967 (20.2)
No	1124 (75.3)	1238 (77.4)	1465 (86.08)	1465 (86.1)
Infecund, *n* (%)^a^				
Yes	11 (0.7)	20 (1.25)	13 (0.8)	44 (0.9)
No	1481 (11)	1580 (98.75)	1689 (99.24)	4750 (0.9)

Women not Pregnant or Infecund (*N*)	1113	1223	1457	3793
Stated desire to use family planning now				
Want to use	844 (75.8)	547 (44.7)	976 67.0)	2367 (34.25)
Do not want to use	154 (13.84)	688 (54.6)	477 (32.7)	1299 (62.4)
Don't Know	112 (10.1)	8 (0.65)	4 (0.27)	124 (3.27)
Missing	3 (0.27)	0 (0.0)	0 (0.0)	3 (0.08)
Current use of family planning, *n* (%)				
Yes	812 (73.0)	377 (30.8)	949 (65.1)	2138 (56.4)
No	269 (24.2)	845 (69.1)	484 (33.2)	1598 (42.1)
Missing	32 (2.9)	1 (0.1)	24 (1.7)	57 (1.5)

Women currently using a method of family planning (*N*)	812	377	949	2138
Current contraceptive method, *n* (%)				
Permanent methods	113 (13.9)	1 (0.3)	436 (45.9)	550 (5.2)
Male sterilization	2 (0.3)	0 (0.0)	0 (0.0)	2 (0.1)
Female sterilization	111 (13.7)	1 (0.3)	436 (45.9)	548 (96.4)
Long‐acting reversible contraception	332 (40.9)	243 (64.5)	112 (11.8)	687 (32.1)
IUD	118 (14.5)	11 (2.9)	72 (7.6)	201 (9.8)
Implant	105 (12.9)	63 (16.8)	25 (2.6)	193 (9.4)
Injectable	113 (13.9)	169 (44.8)	16 (1.7)	298 (13.9)
Short‐acting or on‐demand methods	333 (41.0)	74 (19.6)	354 (37.3)	761 (96.35)
Oral contraceptives	194 (23.9)	45 (11.9)	61 (6.4)	300 (14.0)
Male condom	179 (22.0)	23 (6.1)	292 (30.8)	494 (23.1)
Other	5 (0.7)	13 (3.5)	8 (0.9)	26 (1.22)
Fertility awareness‐based methods	28 (2.5)	54 (14.3)	18 (1.9)	100 (4.9)
Traditional methods	4 (0.5)	9 (2.4)	4 (0.4)	17 (0.8)

^a^ Defined as never menstruated, last menstrual period >2 years prior, or hysterectomy.

Contraceptive desire and use varied considerably by country. Most women in Argentina stated a desire to use a method of family planning (84.1 percent, *n* = 855), and most women were using a method (72.8 percent, *n* = 718). In Ghana, however, only 39.8 percent (*n* = 554) of women stated wanting to use a method, with a much smaller proportion (27.3 percent, *n* = 378) of women using a method. In India, the proportion of women stating a desire to use a method and the proportion of women using a method were largely similar. The contraceptive method mix also varied by country. In India, half of all family planning users were using sterilization (*n* = 437), whereas, in Ghana, 66.3 percent (*n* = 224) of women were using long‐acting reversible methods. Nearly half of all women using a method in Ghana were using injectables (*n* = 169). In Argentina, long‐acting reversible methods (40.8 percent, *n* = 334) were as frequently used as short‐acting methods (41.3 percent, *n* = 338). Overall, 2.6 percent (*n* = 53) of women had unwanted family planning (direct), with the highest proportion in Ghana (4.3 percent, *n* = 16), followed by Argentina (2.5 percent, *n* = 18), and India (2.0 percent, *n* = 19).

**FIGURE 2:**
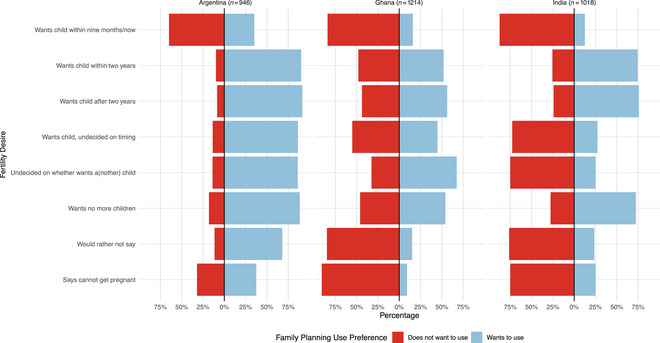
Proportion of women wanting to use contraceptives versus not in relation to fertility desires, among fecund, nonpregnant, and nonsterilized women

Figure 2 illustrates the alignment between a woman's stated desire to use family planning and her stated desire for future pregnancy. While most women who want a pregnancy in the next nine months state that they do not want to use family planning, a considerable proportion across all three countries (17.6 percent, *n* = 47) state that they do want to use family planning. Among women who state wanting a pregnancy within the next nine months (or soon/now), 34.2 percent (*n* = 13) want to use family planning in Argentina, 15.9 percent (*n* = 25) in Ghana, and 12.5 percent (*n* = 9) in India. Across all countries, a woman's stated desire to use family planning is weakly correlated with her desire for another pregnancy within the next nine months (or soon/now) (*r* = 0.38, *p* < 0.001). Among women who stated wanting a child in the next nine to 24 months, our results show that most wanted to use family planning (65.0 percent, *n* = 251). Again, there is considerable variation across countries in the proportion of women who jointly state wanting to use family planning and also desiring a pregnancy in the next nine to 24 months, from 90.4 percent (*n* = 47) in Ghana, 74.4 percent (*n* = 99) in India, and 52.3 percent in Ghana (*n* = 105). Across countries, we find almost no correlation (*r* = 0.06, *p* = 0.01) between a woman's stated desire to use contraception and her desire for a pregnancy after nine months, but within two years. Last, a considerable proportion of women who state wanting no more children also do not want to use family planning. Overall, among women who want no more children, 23.0 percent (*n* = 219) do not want to use family planning, ranging from 46.3 percent (*n* = 57) in Ghana to 11.3 percent (*n* = 47) in Argentina.

**FIGURE 3:**
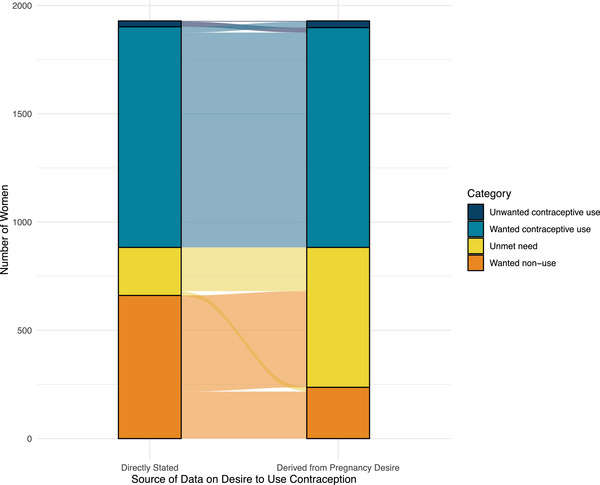
Alluvial plot comparing the proportion of women that fall into the four different categories of family planning need obtained by a woman's directly stated desire to use family planning versus that derived indirectly from future pregnancy desire

Determining “need” for family planning using the direct versus indirect approach also yields different results in terms of the proportion of women who fall into each of the four categories (wanted use, unwanted use, unmet need, and wanted nonuse) (Table 2). Overall, both approaches lead to approximately the same proportion of women being categorized as having unwanted use (indirect: 1.6 percent, *n* = 31 versus direct: 1.4 percent, *n* = 27). However, the women identified by each approach as having unwanted family planning are not the same women (Figure 3). In fact, among the 50 women identified by either approach as having unwanted family planning, only 7.4 percent (*n* = 4) are identified simultaneously by both approaches as having unwanted family planning. Furthermore, the two approaches to categorizing family planning “need” do not consistently identify the same women with unmet need and wanted nonuse. More than half of the women with wanted non‐use who were identified using the direct approach are considered to have unmet need using the indirect approach. Ultimately, 33.5 percent of women in the sample (*n* = 646) were identified as having unmet need for family planning using the indirect approach versus 11.5 percent (*n* = 222, *p* < 0.001) using the direct approach.

**TABLE 2:** Comparison of categories of need by stated desire to use family planning versus need derived from pregnancy desire

	Derived from Pregnancy desire	Directly stated	Difference	*p*‐Value
*N* ^a^	1929	1929		
Wanted use	1019 (52.7)	1023 (52.9)	−0.2	n.s.
Unwanted use	31 (1.6)	27 (1.4)	0.2	n.s.
Unmet need	646 (33.4)	222 (11.5)	21.9	<0.001
Wanted non‐use	237 (12.3)	661 (34.2)	−21.9	<0.001

^a^It includes only women with complete data on both measures.

Using the direct approach, there is little variation in the sociodemographic characteristics of women with wanted versus unwanted family planning (direct) (Table 3). In Argentina, women with unwanted family planning (direct) tend to be older than those with wanted family planning (31.1 years vs. 27.4 years, *p* < 0.05). The only other difference in sociodemographic characteristics between women with unwanted versus wanted family planning was in Ghana. There, women with unwanted family planning (direct) were more likely to report living apart from their spouse than women with wanted family planning (54.6 percent, *n* = 6 vs. 7.9 percent, *n* = 19; *p* < 0.001).

**TABLE 3:** Comparison of sociodemographic and contraceptive use characteristics between women with wanted versus unwanted family planning in Argentina, Ghana, and India

	Argentina			Ghana			India			Total		
	Wanted	Unwanted	*p*‐Value	Wanted	Unwanted	*p*‐Value	Wanted	Unwanted	p‐value	Wanted	Unwanted	*p*‐Value
*N* ^a^	697	18		358	16		929	19		1.984	53	
Age, mean (SD)	27.4 (7.5)	31.3 (7.5)	^*^	28.4 (6.8)	31.4 (7.3)		32.4 (7.1)	31.1 (6.6)		29.9 (7.4)	31.2 (7.0)	
Marital status, *n* (%)												
Currently married/in union	546 (78.3)	12 (66.7)		240 (67.0)	11 (68.8)		900 (96.8)	19 (100.0)		1686 (85.0)	42 (79.2)	
Never married	14 (2.0)	0 (0.0)		108 (30.2)	25 (4)		0 (0.0)	0 (0.0)		122 (6.2)	4 (7.6)	
Widowed/divorced/separated	137 (19.7)	6 (33.3)		10 (2.8)	1 (6.3)		29 (3.1)	0 (0.0)		176 (8.9)	7 (13.2)	
Living apart (if in union), *n* (%)	108 (19.1)	3 (21.4)		221 (90.6)	6 (54.6)	^***^	19 (2.2)	1 (5.3)		150 (8.8)	9 (20.5)	^**^
Wants child within 9 months/now, *n* (%)	9 (1.4)	1 (6.3)		12 (3.4)	1 (6.3)		6 (1.2)	2 (16.7)	^***^	27 (1.8)	4 (9.1)	
Wants child within 2 years/now, *n* (%)	57 (8.3)	2 (11.8)		4 (1.2)	0 (0.0)		428 (46.4)	7 (41.2)		489 (25.0)	9 (18.4)	
Current contraceptive method ^b^, *n* (%)												
Permanent methods	50 (7.2)	4 (22.2)	^**^	1 (0.3)	0 (0.0)		428 (49.7)	7 (36.8)		479 (25.1)	11 (20.8)	
Long‐acting reversible methods	303 (43.5)	5 (27.8)		68.8 (240)	3 (18.8)	^***^	102 (11.9)	10 (52.6)	^***^	645 (33.8)	18 (34.0)	
Short‐acting or on‐demand methods	333 (47.8)	11 (61.1)		115 (33.0)	12 (75.0)	^**^	360 (41.8)	5 (26.2)		808 (42.4)	28 (52.8)	
Male condom‐only	71 (10.2)	6 (33.3)	^**^	5 (1.4)	2 (12.5)	^**^	268 (28.9)	3 (15.8)		344 (17.3)	11 (20.8)	

^a^ Includes nonpregnant, fecund women using family planning

^b^ Percentages of current contraceptive method shown may add to more than 100% due to concurrent method use.

* *p* < 0.05; ** *p* < 0.01; ***p < 0.001.

**FIGURE 4:**
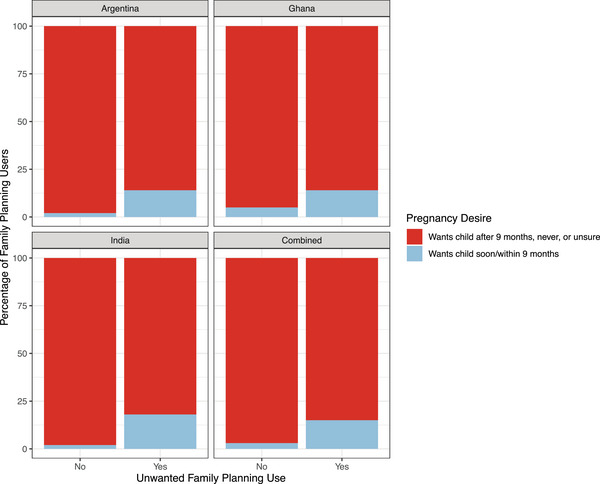
Unwanted family planning use by pregnancy desire

Across all three countries, a greater proportion of women with unwanted family planning reported wanting a pregnancy within the next nine months compared to those with wanted family planning (Figure 4). The largest difference between women with wanted versus unwanted family planning is seen in India, with a significantly higher proportion of women with unwanted family planning who want a child in the next nine months (16.7 percent, *n* = 2, *p* < 0.001) compared to those with wanted family planning (1.6 percent, *n* = 6). While the proportion of women with unwanted family planning who want a child in the next nine months is higher in Argentina (6.3 percent, *n* = 1 vs. 1.4 percent, *n* = 9) and Ghana (6.3 percent, *n* = 1 vs. 3.4 percent *n* = 12) compared to those with wanted family planning, the difference does not reach statistical significance. Even though the proportion of women desiring a pregnancy soon is greater among women with unwanted family planning (direct), it is important to note that overall the majority of unwanted family planning (85.2 percent, *n* = 23) occurs among women who do not want a child within the next nine months.

In addition to pregnancy desire, the method mix varied considerably between women with wanted versus unwanted family planning (direct). Important differences emerged with regard to the use of long‐acting and permanent methods relating to the desired use of contraception. In Argentina, women with unwanted family planning (direct) were more likely to be using permanent methods than women with wanted family planning. In Argentina, 22.2 percent (*n* = 4) of women with unwanted family planning were using permanent methods versus 7.2 percent (*n* = 50) of women with wanted family planning. In Ghana and India, while we found no differences with regard to permanent methods, there were differences in the use of long‐acting reversible methods between women with wanted versus unwanted family planning (direct). In India, women with unwanted family planning (direct) were significantly more likely to be using long‐acting reversible methods (52.6 percent, *n* = 10) compared to women with wanted family planning (11.9 percent, *n* = 102; *p* < 0.001). However, the opposite is true in Ghana, where women with unwanted family planning (direct) were significantly less likely to be using long‐acting reversible contraception when compared to women with wanted family planning. Only 18.8 percent (*n* = 3) of women with unwanted family planning in Ghana reported using long‐acting reversible contraception compared to 68.7 percent (*n* = 241) among women with wanted family planning (*p* < 0.001).

We also find differences between the use of short‐acting and on‐demand methods in relation to whether a woman has wanted versus unwanted family planning (direct). In India, we found that women with unwanted family planning (direct) were less likely to be using short‐acting and on‐demand methods compared to those with wanted family planning. In Argentina and Ghana, women using the male condom as their only method were more likely to report unwanted family planning (direct). In Argentina, 33.3 percent (*n* = 6) of women with unwanted family planning reported using the male condom as their only method compared to 10.7 percent (*n* = 75) among women with wanted family planning (*p* < 0.05). While of a lesser magnitude, in Ghana, 12.5 percent (*n* = 2) of women with unwanted family planning, compared to 1.4 percent (*n* = 5) of women with wanted family planning, reported using only a male condom (*p* < 0.05).

**FIGURE 5:**
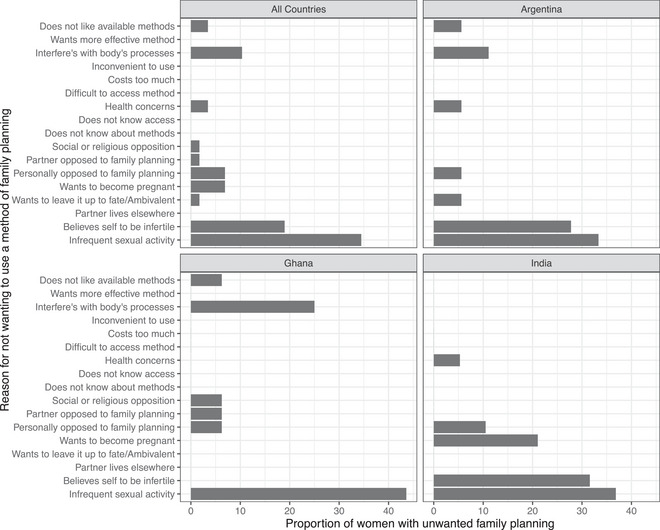
Reasons for unwanted family planning

Women with unwanted family planning (direct) expressed a variety of reasons for not wanting to use a method (Figure 5). Across all three countries, women with unwanted family planning (direct) most often stated that they did not want to use a method due to infrequent sexual activity (37.7 percent, *n* = 20). In Argentina and India, the second most common reason that women with unwanted family planning mentioned was a belief that they thought themselves to be infertile (31.6 percent in India, *n* = 6; 27.8 percent in Argentina, *n* = 5). In Ghana, the second most common reason was the belief that methods interfered with the body's processes (25.0 percent, *n* = 4). Personal or social opposition, concerns related to health effects, or dislike of available methods were also reasons that women with unwanted family planning cited for not wanting to use. Only in India did a desire to become pregnant (21.1 percent, n = 4) feature among the reasons for not wanting to use a method. In Argentina, one woman (5.6 percent) with unwanted family planning (direct) stated that she did not want to use a method due to ambivalence about wanting to become pregnant. None of the women using the male condom as their only method said that the reason for them not wanting to use family planning was because they wanted to become pregnant; the primary reason stated among these women was infrequent sexual activity (72.5 percent, *n* = 8).

## DISCUSSION

Our results suggest that operationalizing unwanted family planning from a person‐centered perspective has important implications for programs and policy. In our study, the lack of concordance between one's desires for a pregnancy and their desired contraceptive use highlights the need for indicators that move beyond using fertility as a proxy for contraceptive desire to encompass a broader sexual and reproductive health agenda. Second, our study adds to the literature on reproductive coercion by finding that most unwanted family planning using the person‐centered approach actually occurs among women who want to prevent a pregnancy, and only a small proportion of unwanted family planning overall is found among women who desire to get pregnant. Finally, we find that a mismatch between women's sexual behavior and method selection may be an important driver behind a significant proportion of unwanted family planning observed in our study.

The lack of alignment between the direct and indirect approaches to estimating the prevalence of unwanted family planning is profound. People's fertility intentions are often complex and abstract (Barrett and Wellings 2002; Rocca et al. 2019; Santelli et al. 2009). The different patterns of family planning use and fertility desires reflect differences across the three countries in this study in terms of their fertility transition. India and Argentina have largely completed their fertility transitions with fertility near or below replacement levels, while Ghana remains mid‐transition with higher fertility rates and larger gaps between women's desired and actual number of children. This reflects Ghana's ongoing demographic transition compared to the more advanced stages reached by India and Argentina. Contraceptive use is lower in Ghana than in Argentina and India, and fertility preferences remain high, which is clearly reflected in our data.

There is a robust literature focused on fertility and contraceptive use that aims to understand the lack of contraceptive use among people wanting to prevent pregnancy. However, research remains limited on understanding the reasons behind contraceptive use when one desires a pregnancy. A study conducted in the United States found that the odds of contraceptive use were lower among women who stated wanting a pregnancy within the next year compared to those who did not; still, nearly half of the women in the study who wanted a pregnancy within the next year were using a method of contraception (Stulberg et al. 2020). In our study, we find that a substantial proportion of women with unwanted family planning express concerns over the interference with their body or other side effects of the methods available. Therefore, despite wanting to prevent a pregnancy, they receive a method that is misaligned with their desires. We argue that this may ultimately undermine their autonomy in choosing the best method for themselves, thus interfering with their ability to prevent a pregnancy when they so desire.

Just as unmet need is meant to be a supply‐side measure that points to shortcomings in the service delivery system to meet demand, perhaps its inverse, the indirect approach to measuring unwanted family planning, could be best utilized in the same way. Senderowicz has previously proposed disaggregating unmet need into supply‐side and demand‐side unmet need to distinguish between unmet need emanating from inadequate services or commodity availability versus a misclassified “need” that reflects a choice not to use contraception (Senderowicz and Maloney 2022). In the case of the indirect approach to measuring unwanted family planning, a similar approach to examining the reasons for the misalignment between one's fertility desires and contraceptive use could help improve programs to enable women to better meet their reproductive goals. Critics of this approach, however, stress that endogeneity between supply and demand side reasons cannot be ruled out (Karra 2024).

In our study, a large proportion of unmet need becomes “wanted nonuse” when re‐categorized with the direct, person‐centered approach. While Karra's concerns over endogeneity still apply, we believe that the simultaneous use of both indicators may point to different contextual challenges and nuances that can support improved family planning programming (Karra 2024). While from a person‐centered perspective, there is no misalignment at the individual level between a woman who does not want to use contraception and her lack of use, even if she wants to prevent a pregnancy. The same holds true for women who want to use contraceptives and are using contraceptives, even if they desire a pregnancy. From a public health perspective, however, such a mismatch warrants a deeper exploration of factors in the socioecological environment contributing to the misalignment. Such factors may occur at a variety of different levels, from a lack of knowledge, a lack of methods with desirable attributes, other service delivery constraints, cost, social norms, women's empowerment and autonomy, etc. Such factors, however, should be addressed at a contextual level—whether that be in providing better information on contraceptives, addressing supply gaps to expand the method mix, or investing in contraceptive technology to design methods that better meet women's desires, while services should be oriented to meet the clients' stated desires. Further research could explore the profile of women who have unmet need according to the traditional measures versus those who have wanted nonuse to identify differences in reasons between women who are not using because they do not want to use versus those who are not using but state a desire to do so.

Some argue that the global health and development community's continued focus on fertility in family planning measurement reflects the field's dark history of coercive programming (Senderowicz 2019). Historically, coercive sterilization has been used in some countries (including in Asia, Europe, and Latin America) as an instrument of population control, without regard for the rights of individuals. This has been particularly well‐documented in India (Patel 2017). Karra and Canning estimate unwanted family planning using the indirect approach among sterilized women in India and find that a substantial proportion of sterilized women in India express regret or were not fully informed of the procedure's permanence (Karra and Canning 2024). In contrast, using our definition of unwanted family planning, our results do not suggest that women with female sterilization in India disproportionately experience unwanted family planning. However, the fact that we find that unwanted family planning using the direct approach is more likely among women in India using long‐acting reversible methods of contraception is concerning, and, given this history, may be suggestive of potential coercion. Several studies have found that some women have been unable to get intrauterine devices (IUDs) and implants removed when they so desire (Amico et al. 2016, 2017), which may emanate from coercive practices on the part of individual providers or at the systems level, where programs do not have adequate resources or capacity for contraceptive removal.

Contraceptive coercion refers to situations where individuals are pressured, manipulated, or forced into using contraceptive methods against their will or without their informed consent. It violates individuals' reproductive rights and autonomy, undermining their ability to make free and informed choices about their own bodies and reproductive health. It can have significant negative consequences on mental, emotional, and physical well‐being and may contribute to distrust in family planning systems. Contraceptive coercion can occur in many different contexts, including relationships, healthcare settings, or through social and cultural pressures and can be implicit or explicit. There are several types of coercion that may be present in the results of this study, though our results cannot definitively point to the role of coercion due to discordance between preferences and behavior. Contraceptive coercion can manifest through physical coercion (forcing someone to use contraception through threats or physical force), psychological coercion (pressuring someone emotionally or psychologically, such as threatening to leave a partner or accusing them of not being responsible if they do not use contraception), or institutional coercion (which includes practices by healthcare providers or policies that pressure individuals, particularly vulnerable groups into using contraception in general, or specific methods, such as sterilization or long‐acting contraceptives, without their full consent or understanding). For example, a health care provider could pressure a woman to continue using a long‐acting reversible method (psychological coercion), refuse to remove it because they believe the woman should continue using it (physical coercion), or because the necessary supplies for removal were not available to the provider due to implicit or explicit pressure to prioritize continuation of long‐acting methods (institutional coercion). At the same time, a provider who feels most comfortable counseling on long‐acting methods when compared to other methods without any other underlying motivation is more likely an issue of training and service quality rather than coercion, even though it may manifest in a similar outcome. Existing research is limited on reproductive coercion perpetrated at the systems and structural level. Our results underscore the importance of better understanding how attributes of the health system may directly or indirectly undermine women's autonomous contraceptive choice by failing to consider their own specific life circumstances.

At the same time, coercion may not be the only reason for discordance between contraceptive preference and use. Discordance may simply be the result of changing preferences over time coupled with inertia. For example, women with unwanted family planning in our study using a long‐acting reversible method may want to remove it, but have not gone back to a provider to do so. Discordance could also mean that some women are unhappy with their method, or unhappy with the entire range of contraceptives available, or that they simply wish they did not have to use a contraceptive method to control their fertility. Important to understanding the possible role of contraceptive coercion in our study results is that women were able to express ambivalence in their desire to use a method. Women who responded with a response of “I don't know” to the question about whether they wanted to use a contraceptive were not considered to have unwanted use. Our analysis was conducted among women who expressed clear preferences in one direction or another, which may limit some of the preference‐related reasons for unwanted use.

The most common reason we found among women with unwanted family planning was a lack of sexual activity. Sexual activity among married women should not be assumed (Gausman et al. 2025), and sexual infrequency may in fact be common. In settings where the provision of long‐acting reversible methods is common, improved counseling that includes a discussion of a person's sexual behavior may help a woman choose a method that is better aligned with her needs. Interestingly, in Ghana, while we did not see increased unwanted family planning among women using long‐acting reversible methods, our results show that almost half of the women with unwanted family planning in Ghana reported infrequent sexual activity or living apart from their partner. Providers are often underprepared to have discussions with clients about their sexual health and sexual behavior, as services are often oriented to focus specifically on reproduction in counseling sessions (Wood et al. 2022; Beebe et al. 2021). Adding to this, the standard definition of unmet need makes assumptions about women's fertility. In settings where testing is not available to establish male or female fertility, an individual's own beliefs about their own, or their partner's, fertility should be valued.

One of the most common reasons for unwanted family planning use in the study is related to women's beliefs that they were infertile. Valuing women's personal beliefs related to infertility raises several important questions about agency and choice in contraceptive decision‐making. Some of these women may use contraception despite their desire not to risk a mitigation strategy if they are not fully certain about their infertility. Global family planning investment has traditionally emphasized contraception as a means to manage fertility, with comparatively less attention to infertility testing and treatment in resource‐constrained settings. Yet, the differential allocation of resources may reflect the historical context of population policies that prioritized fertility reduction in low‐ and middle‐income countries. The historical emphasis on fertility prevention, rather than supporting the full spectrum of reproductive choices, including fertility enhancement, therefore, may be an ongoing example of reproductive coercion as it relates to assumptions about populations that should reproduce.

Surprisingly, we found a relatively large proportion of unwanted family planning occurring to women using the male condom as their only method. A similar result was found in Canning and Karra's analysis of unwanted family planning (indirect), who posit that in this case, women may want the protective benefit of the male condom against HIV and other STIs, but not the contraceptive benefits of the method. In our study, none of the women with unwanted family planning (direct) who were using the male condom as their only method said that their lack of desire to use a contraceptive method was because they wanted to become pregnant. Thus, our data suggest that the contraceptive benefits of the male condom are also desired among these women, yet they state not wanting to use a method. While there is considerable research documenting decreased pleasure and sexual enjoyment resulting from male condom use among men, few studies have examined the impact of the male condom on women's sexual experiences and satisfaction (Wood, Karp, and Zimmerman 2020). More research into understanding partner dynamics, experiences, and sexual enjoyment in negotiating condom use within the context of unwanted family planning would be useful.

This study has several strengths and limitations worth noting. In terms of strengths, our study is one of the few to examine unwanted family planning from a person‐centered perspective using women's directly stated desire for contraception. Our data come from a large and representative sample across three diverse countries. In terms of limitations, our analysis is somewhat limited due to the low prevalence of unwanted family planning across the study sites. As a result, the main challenge in interpreting our results is the increased risk of Type II error, or failing to detect a true difference when one exists. Therefore, the null results presented in our study should be interpreted with caution and may warrant further investigation with larger samples. Conversely, the small number of observations with unwanted family planning use may reduce statistical power, but does not inherently increase Type I error risk. Rather, a Type I error relates to the probability of falsely rejecting a true null hypothesis, which is controlled by our chosen significance level. Most of our results have *p*‐values below 0.05, which indicates statistical significance at the conventional alpha level. However, the prevalence of unwanted family planning we find is surprisingly similar to other studies on unwanted family planning that define it differently (Canning and Karra 2023). Further, as we use secondary data, we were unable to explore in more depth the reasons behind women's lack of desire for contraceptives or the reasons why they were using contraception against their wishes.

## CONCLUSIONS

Our results echo previous calls on the global health community to be more explicit in measurement frameworks by clarifying the motivations behind the field's continued use of fertility preferences as a proxy for contraceptive desire or need. More research on examining different forms of reproductive coercion from a person‐centered perspective across the social‐ecological system would better ground the field of global family planning in both human rights principles but also within the post‐Cairo agenda of comprehensive sexual and reproductive health.
